# Hepatobiliary Disorders in Celiac Disease: An Update

**DOI:** 10.4061/2011/438184

**Published:** 2010-11-14

**Authors:** Kaushal K. Prasad, Uma Debi, Saroj K. Sinha, Chander K. Nain, Kartar Singh

**Affiliations:** ^1^Department of Superspeciality of Gastroenterology, Postgraduate Institute of Medical Education and Research, Chandigarh 160 012, India; ^2^Division of GE Histopathology, Department of Superspeciality of Gastroenterology, Postgraduate Institute of Medical Education and Research, Chandigarh 160 012, India; ^3^Department of Radiodiagnosis, Government Medical College & Hospital, Sector 32, Chandigarh 160030, India

## Abstract

This communication reviews recent literature and summarizes hepatobiliary abnormalities that may complicate the clinical course of celiac disease. A wide spectrum of hepatobiliary diseases has been described, including asymptomatic elevations of liver enzyme levels, nonspecific hepatitis, nonalcoholic fatty liver disease, and autoimmune and cholestatic liver disease. Moreover, in the majority of patients, liver enzyme levels will normalize on a gluten-free diet. In addition, celiac disease may be associated with rare hepatic complications, such as hepatic T-cell lymphoma. Because many celiac patients do not have overt gastrointestinal symptoms, a high index of suspicion is required. Simple methods of detecting celiac disease such as serum antibody tests help in the early identification of the disease, thus preventing serious complications of the disorder. The IgG DGP antibody test and IgA tTG antibody test used in combination are an excellent screening test for suspected cases of celiac disease.

## 1. Introduction

Celiac disease (CD), or gluten-sensitive enteropathy (GSE), is an autoimmune disorder characterized by reversible small bowel mucosal inflammation with villous atrophy affecting patients with a specific genetic predisposition (HLA DR3-DQ2 and HLA DR4-DQ8). The mucosal lesion develops after ingestion of dietary gluten and recovers when gluten-containing cereals, wheat, rye, and barley, are withdrawn from the diet [1]. In untreated CD, the characteristic abnormalities in the small bowel mucosa are villous atrophy, crypt hyperplasia, and an increased density of inflammatory cells in the epithelium and lamina propria [2–4]. The small bowel lesion of CD is a dynamic process whereby mucosal damage to the small intestine develops in three subsequent phases: (a) infiltrative phase, characterized solely by an increased number of intraepithelial lymphocytes; (b) hyperplastic phase, characterized by crypt hypertrophy; (c) destructive phase, which is characterized by progressive villous atrophy ultimately leading to the flattening of the mucosa [5]. 

Abnormal immune response to gliadin, genetic, and environmental factors plays a role in the pathogenesis of CD. Infectious agents have been implicated in the pathogenesis of many autoimmune disorders. Transient infections or increased permeability of the mucosa may facilitate disease onset induced by the uptake of gluten peptides into a microenvironmental milieu in the small intestinal mucosa [6]. An environmental factor, such as an infectious agent, is thought to precipitate the disease via various pathogenic mechanisms, such as molecular mimicry, resulting in modulation of the host's immune tolerance [7]. Recently, two patients with onset of CD after resolution of acute hepatitis B virus (HBV) infection have been reported in the literature [6]. Celiac disease has also been described in association with hepatitis C virus (HCV), rotavirus, adenovirus 12, and astrovirus as another immunologic manifestation of this infectious disease [8–11]. On the contrary, Plot et al. (2009) examined the association between serological evidence of past infection with *Toxoplasma gondii*, rubella virus, cytomegalovirus, *Treponema pallidum*, and Epstein-Barr virus and the coexistence of CD. The results implied that certain infections may generate an immunological environment that disfavours future appearance of certain autoimmune conditions such as CD [12]. Hence, understanding the relationship between infectious agents and autoimmune disorders is of importance that may assist in prediction, early diagnosis, and perhaps also the prevention of CD [7].

Population-based screening studies have shown that CD is very common and affects about one in 120 [13]. The diagnosis of CD is easily overlooked as patients can present with mild or atypical symptoms or the condition can even be clinically silent. Patients may present with only subtle, if any, symptoms [14], which is the main reason why the disease is highly underdiagnosed in India and elsewhere. In recent years, recognition of CD has been substantively improved with increasing awareness for CD amongst health care professionals and partly as a result of more modern serological assays for screening. The advent of serological methods for the detection of antibodies to gliadin, endomysial, and tissue transglutaminase has enabled large-scale screening for CD, thus preventing serious complications of the disorder. The most common serological tests for the screening of CD are the indirect immunofluorescence (IFA) method of detecting endomysial antibodies (EmA) and ELISA methods of detecting antibodies to tissue transglutaminase (tTG) and gliadin [15, 16]. Because of the limited sensitivity and specificity of gliadin antibody assays, use of this method is more limited than EmA and tTG. Reliance on EmA and tTG immunoassays, however, may allow cases of IgA-deficient CD to go undetected [17]. Both immunoassays may be of limited utility detecting IgG antibodies. IgG gliadin antibody tests are useful in establishing diagnosis of CD in IgA deficient patients. More recently, a new generation of promising assays detecting the presence of deamidated synthetic peptides of gliadin (DGP) has shown very high diagnostic performance equivalent to conventional tests [18, 19]. The IgG DGP and IgA tTG in combination are an excellent screening algorithm for suspected cases of CD [19].

Although most symptomatic patients have symptoms related to the gastrointestinal tract, many extraintestinal manifestations have been described. The target organs include endocrine system [20–22], kidney [23, 24], skin [25], nervous system [26], and reproductive systems [27]. A number of hepatobiliary tract and pancreatic disorders have also been documented in patients with CD [28]. Some of these have been hypothesized to share common genetic factors or have a common immunopathogenesis, such as primary biliary cirrhosis (PBC), primary sclerosing cholangitis (PSC), and autoimmune forms of hepatitis or cholangitis [28]. Other hepatic changes in CD that may be associated with increased intestinal permeability could result in translocation of gut bacteria, kupffer cell stimulation, and production of tumor necrosis factor-*α* (TNF-*α*), proinflammatory cytokine, and reactive oxygen species, resulting in nonalcoholic steatohepatitis (NASH). It is also hypothesized that the malnutrition in CD might lead to chronic deficiency of a lipotropic factor (e.g., choline), with an associated pyridoxine deficiency, resulting in hepatic steatosis [29]. In addition, CD may be associated with rare hepatic complications, such as hepatic T-cell lymphoma [28]. Finally, pancreatic abnormalities may be caused by CD or coexist with CD. Both endocrine and exocrine functions of the pancreas may be substantially changed in CD. We review the literature pertaining to hepatobiliary abnormalities that may be seen in association with celiac disease.

## 2. Hepatobiliary Disorders

Hepatic changes in patients with CD were initially described by Pollock in 1977 [30]. Since then the relationship between hepatobiliary dysfunction and CD is well established, ranging from asymptomatic or cryptogenic hypertransaminasemia to chronic liver disease (CLD) [31]. Thus, the hepatologist needs to consider CD in the differential of abnormal liver blood tests and to be aware of the clinical implications of this frequent disease in patients with liver disorders. The etiology is varying and multifactorial, such as genetic predisposition, precocity, and duration of exposure to gluten that may influence reversibility of liver damage [32]. 

### 2.1. Asymptomatic Elevations of Liver Enzyme Levels

Transaminases (ALT (alanine aminotransferase) and AST (aspartate aminotransferase)), *γ*-glutamyl transpeptidase (*γ*-GT), and alkaline phosphatase (ALP) are classically termed as liver enzymes; however, they can be found in almost every organ. Elevated levels of the transaminases ALT and AST are signs of disturbed permeability of the cells, in which these enzymes can be found. In contrast to ALT, which is mainly liver specific, the AST is found in other organs as well, for example, heart and skeletal muscle [33]. As such hypertransaminasemia has been observed in up to 40% of untreated celiac patients [34]. Celiac disease at diagnosis may present in 5%–10% of patients as an asymptomatic or cryptogenic hypertransaminasemia, as an expression of a mild liver dysfunction with a histological picture of nonspecific reactive hepatitis (celiac hepatitis) which frequently disappears after gluten suppression [32, 35–37]. At a mild elevation of liver enzymes, reevaluation is recommended; however, if an elevation persists and is suspicious for a liver disease, a specific workup is necessary [33].

### 2.2. Nonalcoholic Fatty Liver Disease

Nonalcoholic fatty liver disease (NAFLD) refers to a spectrum of diseases of the liver ranging from simple steatosis (i.e., fatty infiltration of the liver) to nonalcoholic steatohepatitis (i.e., steatosis with inflammation and hepatocyte necrosis (NASH)) to cirrhosis. Nonalcoholic fatty liver disease is the most common cause of elevated liver enzymes in adults and the most common cause of cryptogenic cirrhosis, which is cirrhosis that cannot be explained by hepatitis, alcohol abuse, toxin exposure, autoimmune disease, congenital liver disease, vascular outflow obstruction, or biliary tract disease [38]. 

Celiac disease has been reported in 4%–13% of cases with NASH [39–41]. The etiopathogenesis of hepatic steatosis in CD is uncertain. It has been suggested that malabsorption in CD might lead to chronic deficiency of a lipotropic factor (e.g*.,* choline), with an associated pyridoxine deficiency, and hepatic steatosis might occur [29]. It is plausible that CD, like small intestinal bacterial overgrowth (SIBO), can be associated with increased intestinal permeability [42]. The increased intestinal permeability could result in translocation of gut bacteria, kupffer cell stimulation, and production of tumor necrosis factor-*α* (TNF-*α*), proinflammatory cytokine, and reactive oxygen species, resulting in NASH. Further studies are warranted to define the precise pathogenetic mechanism or mechanisms for NAFLD in CD.

### 2.3. Autoimmune Hepatobiliary Disorders

Autoimmune hepatobiliary disorders are frequently associated with CD, but they might remain undiagnosed because of lack of symptoms, absence of liver-specific autoantibodies, or misdiagnosis of celiac hepatitis. Acute hepatitis in celiac patients should induce one to suspect an autoimmune origin [43]. Patients with end-stage autoimmune liver disease, especially those who are HLA-DQ2 or HLA-DQ8 positive, had a high prevalence of CD-associated antibodies [44]. Indeed, CD has been found in 3%–7% of patients with PBC, in 3%–6% with autoimmune hepatitis (AIH), and in 2%-3% with PSC. Differently from cryptogenic liver injury, autoimmune liver dysfunction, found in CD, does not usually improve after a gluten-free diet (GFD) [35]. Since CD is common among patients with autoimmune liver disease, screening autoimmune liver disease patients for CD is indicated. Although the magnitude of benefit from a GFD in reversing autoimmune liver disease in patients with CD is controversial, it may reduce the risk of further complications of CD [45]. 

#### 2.3.1. Autoimmune Hepatitis and Cholangitis

Although AIH has been reported in only a limited number of case reports and population-based studies, the prevalence of CD has been found in 3%–6% with AIH [35, 45]. Conversely, patients with CD have a sixfold greater risk of AIH compared to the general population [46]. The two forms of AIH with different serological and clinical features can be found in association with CD. Indeed, the two well-defined forms of AIH, found in CD, are AIH type 1, which is characterized by positivity for ANA/SMA and affects much more frequently adult patients, and AIH type 2, characterized by LKM and/or LC1 antibody positivity, usually confined to childhood CD [47, 48]. Seronegative AIH has also been reported with CD [49]. In CD with AIH, GFD plus immunosuppressants determines a high remission rate. When clinical remission is reached, a prolonged immunosuppressive regimen induces a high sustained remission rate after treatment withdrawal [50]. 

Autoimmune cholangitis (antimitochondrial antibody-negative PBC) is a rare chronic cholestatic liver disease. CD should be considered in all patients diagnosed with autoimmune cholangitis as a GFD may avoid the need for immunosuppressive therapy in affected patients [51].

#### 2.3.2. Primary Sclerosing Cholangitis

An association between CD and PSC was first described in1988 by Hay et al. in a report of three patients with PSC later diagnosed with CD [52]. Thereafter, few other cases were reported [53, 54]. It has been difficult to show good evidence for a response of the PSC to a gluten-free diet.

#### 2.3.3. Primary Biliary Cirrhosis

The association between CD and PBC was first described in four patients by Logan et al. [55].   Numerous case reports followed formal epidemiological studies to determine the prevalence of PBC in patients with CD [56]. Although an association between PBC and CD has been reported, there are still conflicting data concerning this issue [57]. Considering the malabsorption, weight loss and osteoporosis are clinical manifestations common to both entities, screening for CD in PBC and vice versa, for antimitochondrial antibodies are advisable in adult CD patients for early diagnosis and institution of specific therapeutic measures [57].

### 2.4. Miscellaneous Hepatobiliary Disorders in Celiac Disease

Celiac disease can present great clinical heterogeneity. Its association with a series of intestinal and nonintestinal diseases, whether immunologically mediated or otherwise, presents a higher than normal frequency. Celiac disease has increasingly been reported with a variety of other liver diseases.

#### 2.4.1. Hemochromatosis

Celiac disease and hereditary hemochromatosis (HH) are genetic disorders with opposite effects on iron absorption and probably on the absorption of some other divalent metals including copper [58]. Hereditary hemochromatosis is the most common autosomal recessive disease in the Caucasian population and is characterised by an iron overload state. Celiac disease, or GSE, on the other hand is frequently associated with iron deficiency anemia. The concomitant presence of HH and CD is rare, and susceptibility to both is associated with genes in the major histocompatibility complex (MHC) on the short arm of chromosome 6 [59, 60]. However, their MHC association is quite distinct. CD is associated with the HLA-DQ2 allele as well as non-HLA genes, whereas HH is associated with the HLA-A3 and B7 alleles of the MHC region [61]. It is not clear whether there is a genetic relationship between the two conditions or their association merely coincidence based on their higher frequencies in the general population. 

Occult CD may compensate for increased divalent-metal transporter 1 (DMT1) expression in a specific subset of individuals with homozygous C282Y mutations in the hemochromatosis (HFE) gene, thus contributing to the low penetrance of HH [62]. On the other hand, HFE mutations do not constitute a protective factor against the development of iron deficiency, which seems to be mainly determined by the severity of the intestinal lesions [63].

#### 2.4.2. Gallstone Diseases

Impaired gallbladder motility has been reported also in patients with CD in relation to reduced secretion of enteric hormones and/or decreased gallbladder sensitivity to them. In particular, untreated celiacs showed low postprandial cholecystokinin and increased fasting somatostatin levels [64]. In spite of these physiological alterations, there does not appear to be a significant predisposition to gallstones in CD except for elderly celiac [65].

#### 2.4.3. Hepatic Vein Obstruction

The association of Budd-Chiari syndrome (BCS) and CD is rare and defined by obstruction of the hepatic vein. All but one of the reported cases had thrombosis of hepatic veins, and one patient had membranous obstruction of inferior vena cava (IVC) [66–68]. Celiac disease should be considered especially in the event of BCS and malabsorption. The long-term outcome is favorable with a GFD and antivitamin K treatment [69]. A percutaneous balloon angioplasty was performed, with satisfactory outcome in case of membranous obstruction of the inferior vena cava [68].

#### 2.4.4. Noncirrhotic Portal Hypertension

Nodular regenerative hyperplasia (NRH) of the liver is a rare disorder that is characterized by a nodular transformation of the hepatic parenchyma without fibrous septa between the nodules, is often associated with connective tissue disorders, hematological malignancy, or drugs, and is a cause of noncirrhotic portal hypertension [70, 71]. Antiphospholipid antibodies (aPL) are a heterogeneous group of circulating autoantibodies including anticardiolipins found in the sera of healthy subjects and patients with autoimmune and infectious diseases. IgA anticardiolipin antibodies were reported recently in celiac patients with NRH [72, 73]. 

Idiopathic portal hypertension (IPH) or noncirrhotic portal fibrosis (NCPF) is a disorder generally classified as a noncirrhotic portal hypertension of unknown etiology and is clinically characterized by portal hypertension, splenomegaly, and pancytopenia [74, 75]. Idiopathic portal hypertension is a heterogeneous and multifactorial disorder with a potential genetic contribution, seen most often in the Indian subcontinent and in East Asia [76]. The association of CD with IPH has been recently reported in the literature [77, 78]. The improvement of portal hypertension with a GFD implicates a causal relationship between portal hypertension and increased inflammatory reactions in CD [79]. The coexistence of CD and IPH suggests that there may be an immunological link between these two conditions [78]. Therefore, the testing for CD in patients with IPH is warranted.

#### 2.4.5. Hepatic Malignancies

Celiac disease is associated with intestinal lymphoma and other forms of cancer, especially adenocarcinoma of the small intestine, of the pharynx, and of the esophagus. Enteropathy-associated T-cell lymphoma (EATL) is a rare form of high-grade, T-cell non-Hodgkin lymphoma (NHL) of the upper small intestine that is specifically associated with CD. Enteropathy-associated T-cell lymphoma derives from a clonal proliferation of intraepithelial lymphocytes (IELs) and is often disseminated at diagnosis. Extraintestinal presentations are not uncommon in the liver, spleen, thyroid, skin, nasal sinus, and brain [80]. Lymphoma in the liver is apparently secondary to intestinal lymphoma, complicating celiac disease [30]. In general, involvement of the liver in CD patients with lymphoma is limited and overshadowed by the clinical course of the intestinal disease. The outlook of EATL is poor. Hepatocellular carcinoma (HCC) in one patient and hepatosplenic lymphoma, a rare type of peripheral T-cell lymphoma with rearrangement of the *γδ*T-cell receptor, have been reported [30, 81, 82].

#### 2.4.6. End-Stage Liver Disease

Although rare, severe hepatic damage or failure can develop in association with CD [31]. In a few cases, when CD is diagnosed, a more severe liver injury, characterized by a cryptogenic chronic hepatitis or liver cirrhosis, was present [35]. Celiac disease is up to 10 times more frequent among patients with CLD [83]. Severe liver failure and decompensated cryptogenic liver cirrhosis are potentially treatable with institution of a GFD. Dietary treatment may prevent progression to hepatic failure, even in cases in which liver transplantation is considered [84]. Therefore, all patients with cryptogenic cirrhosis should be screened for CD-related antibodies before proceeding to a liver transplant.

Increased intestinal permeability, SIBO, kupffer cell abnormalities, direct toxicity of gliadin peptides, or a gliadin-stimulated immunopathological process may have a role to play in the progression of CLD among celiac patients [83, 84]. The patients with CLD have a higher likelihood of false positive results regarding anti-tTG antibodies, which is due, in some instances, to increased immunoglobulins concentrations and, in most individuals, to the role of transglutaminase in liver fibrosis mechanisms [85]. The specificity and sensitivity of tTG assays have been questioned, and several publications describe positive tTG antibody results in autoimmune diseases other than CD, while the samples are negative for EmA [86–88]. Furthermore, the finding of false positive results for anti-tTG antibodies is a relevant problem for antibodies to guinea pig tTG, whereas the number of false positives is far much lower for antibodies to recombinant human tTG, the usually routine test for CD screening. More recently, a new generation of promising assays detecting the presence of deamidated synthetic peptides of gliadin (DGP) has shown very high diagnostic performance equivalent to conventional tests [18, 19]. The combination of two assays makes diagnostic accuracy higher. Therefore, a serologic screening by combining tTG IgA with DGP IgG or EmA testing could be considered as the best initial test for CD [19].

## 3. Conclusions

In conclusion, a number of hepatobiliary disorders have been documented in patients with celiac disease. Consequently, newly onset celiac disease should be rigorously checked for asymptomatic hepatic damage and conversely, any cryptogenic liver disorder, including end-stage liver disease, should be investigated for celiac disease. Presently, it is difficult to establish if the two main kinds of hepatic injury (cryptogenic and autoimmune) found in celiac disease are discrete entities with a different pathogenesis or if they are an expression of the same disorder, where genetic factors and the duration of gluten exposure may determine the severity and the pattern of hepatic injury. Several communications of isolated cases of rare hepatobiliary diseases, which probably, only reflect a fortuitous association with celiac disease, have been cited in the literature.
